# Protonation Dynamics on Lipid Nanodiscs: Influence of the Membrane Surface Area and External Buffers

**DOI:** 10.1016/j.bpj.2016.03.035

**Published:** 2016-05-10

**Authors:** Lei Xu, Linda Näsvik Öjemyr, Jan Bergstrand, Peter Brzezinski, Jerker Widengren

**Affiliations:** 1 Experimental Biomolecular Physics, Department of Applied Physics, KTH-Royal Institute of Technology, AlbaNova University Center, Stockholm, Sweden; 2 Department of Biochemistry and Biophysics, Arrhenius Laboratories for Natural Sciences, Stockholm University, Stockholm, Sweden

## Abstract

Lipid membrane surfaces can act as proton-collecting antennae, accelerating proton uptake by membrane-bound proton transporters. We investigated this phenomenon in lipid nanodiscs (NDs) at equilibrium on a local scale, analyzing fluorescence fluctuations of individual pH-sensitive fluorophores at the membrane surface by fluorescence correlation spectroscopy (FCS). The protonation rate of the fluorophores was ∼100-fold higher when located at 9- and 12-nm diameter NDs, compared to when in solution, indicating that the proton-collecting antenna effect is maximal already for a membrane area of ∼60 nm^2^. Fluorophore-labeled cytochrome *c* oxidase displayed a similar increase when reconstituted in 12 nm NDs, but not in 9 nm NDs, i.e., an acceleration of the protonation rate at the surface of cytochrome *c* oxidase is found when the lipid area surrounding the protein is larger than 80 nm^2^, but not when below 30 nm^2^. We also investigated the effect of external buffers on the fluorophore proton exchange rates at the ND membrane-water interfaces. With increasing buffer concentrations, the proton exchange rates were found to first decrease and then, at millimolar buffer concentrations, to increase. Monte Carlo simulations, based on a simple kinetic model of the proton exchange at the membrane-water interface, and using rate parameter values determined in our FCS experiments, could reconstruct both the observed membrane-size and the external buffer dependence. The FCS data in combination with the simulations indicate that the local proton diffusion coefficient along a membrane is ∼100 times slower than that observed over submillimeter distances by proton-pulse experiments (*D*_*s*_ ∼ 10^−5^cm^2^/s), and support recent theoretical studies showing that proton diffusion along membrane surfaces is time- and length-scale dependent.

## Introduction

Proton gradients across membranes generated by membrane-bound proton pumps and subsequently used in, e.g., ATP synthesis, transmembrane transport, and motility, is a central part of cellular energy turnover. Yet, the detailed mechanisms for the proton transport in these membrane proton circuits remain unclear (1). For specific membrane-bound proton pumps such as cytochrome *c* oxidase (Cyt*c*O) or bacteriorhodopsin, protonation rates have been found that are significantly faster than if the rates were purely diffusion-limited (2, 3, 4, 5). These findings indicate that in membranes the protonation is facilitated by surface-accessible negative and buffering residues in the proteins, located near the entry points of their proton-conducting pathways (6, 7, 8). Moreover, theoretical studies indicate that the membrane-water interface surrounding the membrane-bound proteins can also play an important role for enhancing the uptake of pumped protons (5, 6, 9, 10). The interface can form a proton-collecting antennae with a radius *R*_PCA_ related to how far a proton generated at the membrane surface can diffuse from its site of generation before the probability to find a proton equals the average value on the surface (11). Thereby, the proton-uptake rate can be far higher than that given by proton diffusion in water and the physical cross section of the proteins. From light-induced proton-pulse studies, it has been found that protons released at planar purple (12) and artificial membranes (13, 14) display diffusion coefficients along the membrane-water interfaces (*D*_*s*_) as high as 5 × 10^−5^ cm^2^/s. This can result in local coupling of the proton flow between spatially separated proton pumps and consumers, with the uptake taking place before the protons escape away from the membrane surface into the bulk phase, and in effective *R*_PCA_ as large as tens of micrometers (15).

An alternative to proton-pulse studies is offered by fluorescence correlation spectroscopy (FCS). FCS analyses dynamic processes of fluorescent molecules in a confocal detection volume, excited by a focused laser beam (16). A broad range of molecular dynamic processes can be studied, as long as they reflect themselves via fluctuations in the detected fluorescence intensity. By fluorescence intensity fluctuations studied via FCS, blinking rates and the fractions of fluorescent and nonfluorescent fluorophores can be determined. Monitoring a low number of pH-sensitive fluorophores at a time as they diffuse through the confocal detection volume, free in solution, or labeled to lipids or proteins, it is also possible to determine the proton exchange to and from the fluorophores in their specific local environment at steady state (17, 18, 19). Using this approach, proton uptake rates of dyes bound to a lipid within small (30-nm diameter) 1,2-dileoyl-*sn*-glycero-3-[phospo-*rac*-(1-glycerol)] (DOPG) liposomes (20, 21), or to the surface of Cyt*c*O, subsequently inserted into the same type of liposomes (22), have been studied. These FCS experiments could directly demonstrate proton-uptake rates more than two orders-of-magnitude higher than for free dyes in aqueous bulk solution. However, *D*_*s*_ on the liposome surface was estimated to be only ∼2 × 10^−7^ cm^2^/s (20), i.e., two orders-of-magnitude smaller than the range of *D*_*s*_ determined by proton-pulse experiments. Such a low *D*_*s*_ in comparison to that in bulk water (*D*_*w*_ = 9.3 × 10^−5^cm^2^/s (23)) is still compatible with strongly enhanced proton-uptake rates because the proton concentrations at the membrane surface may be significantly higher than those in the bulk solution (20). Although comparatively small, this *D*_*s*_ is still a factor of 2–3 higher than the diffusion coefficients for the lipids themselves (24), which indicates that the proton exchange is not only controlled by lipid diffusion.

The apparently inconsistent range of *D*_*s*_ reported from proton-pulse (12, 13, 14, 25), FCS (20, 21, 22), and nuclear magnetic resonance (26) experiments has recently been addressed in theoretical simulation studies (27, 28). These studies suggest that proton diffusion on lipid membranes is anomalous, and that *D*_*s*_ is time- and length-scale dependent, with a short-range, subdiffusive regime and a long-range, superdiffusive regime. These studies also predict trapping of hydrated excess protons at the membrane surface, capable to yield 100-fold higher proton concentrations at the membrane surface rather than the bulk water, as inferred from FCS experiments (20, 21, 22). The long-range (tens of micrometers) proton translocation along biological membranes has been attributed to coupled bulk diffusion, with the protons desorbing and readsorbing at the surface thousands of times during their translocation (1). As predicted from a general form of Fick’s law, coupled bulk diffusion and the Grotthuss mechanism can make *D*_*s*_ for this mode of long-range proton translocation almost as prominent as in bulk water, while membrane-bound and bulk buffers significantly reduce *D*_*s*_ in proportion to the fraction of protonated buffer molecules and by their limited diffusivity compared to *D*_*w*_ (23, 29). These buffer effects, as also observed by proton-pulse experiments (13, 14, 24, 30), lead to significantly smaller *R*_PCA_ values, already at mM buffer concentrations (11, 15, 31).

Given that both *D*_*s*_ and the exchange of protons between the membrane surface and the bulk appear to be length- and time-scale-dependent (27, 28), it is of interest to investigate how *R*_PCA_ and effects of external buffers may differ on a local level, compared to when observed for long-range proton translocation over tens of micrometers. In our previous FCS studies based on dye-labeled liposomes (20, 21, 22), variation of the size of the liposomes may be expected to yield a difference in the proton exchange rates of the dyes and thereby make it possible to quantify how the membrane size influences *R*_PCA_. However, it is difficult to make the vesicles smaller than 30 nm in diameter, i.e., with a membrane surface area of ∼2800 nm^2^. In addition, it is difficult to separate any effects of membrane curvature from those attributed to the size of the surrounding membrane area. In the FCS studies, no buffers were added into the bulk solution (20, 21, 22). However, for buffers acting on free fluorescein in a three-dimensional aqueous solution (18), or with both the fluorophore and the buffer molecules confined to a two-dimensional membrane (2D) (20), a linear dependence of the local proton exchange rate to the buffer concentration was observed.

Here, based on steady-state FCS measurements, we investigated the effect of the membrane size on the local proton exchange using planar membrane nanodiscs (NDs) (32) with diameters of 9 nm (area ∼60 nm^2^) or 12 nm (area ∼110 nm^2^), with their sizes determined by membrane-scaffold proteins surrounding the discs. The NDs were labeled by introducing a lipid molecule with an individual pH-sensitive fluorescein dye attached to it. Alternatively, to study the effect of the membrane area also for proton exchange close to membrane proteins, and to further restrict the lipid membrane area in the NDs, fluorescein-labeled Cyt*c*O was inserted into nonlabeled NDs. Further, we studied the influence of buffers on the local proton exchange, by measurements on NDs at different concentrations of buffers added to the aqueous bulk solution.

The experiments showed that a membrane area as small as a few nanometers in radius is sufficient to effectuate a local proton collection antenna effect. With buffers added in the solution, a buffer concentration dependence was found, with the proton exchange rates first decreasing with higher buffer concentrations and then, at millimolar buffer concentrations, increasing. Monte Carlo simulations of the proton exchange at the membrane-water interface indicate that the observed local membrane-size and external buffer dependence can be explained by a simple kinetic model, as a competition between two processes: proton translocation along the membrane surface; and buffer-mediated proton exchange between the membrane surface and the bulk. With increasing buffer concentrations and smaller membrane areas, the surface-bulk exchange dominates. At the highest buffer concentrations, the proton-collecting antenna effect disappears, but due to an increase in the direct proton exchange between the bulk and the fluorophore there is an overall increase of its proton exchange.

## Materials and Methods

### Growth, purification, and labeling of wild-type cytochrome *c* oxidase

*Rhodobacter sphaeroides* was grown in Sistrom media, and His-tagged wild-type Cyt*c*O was purified using a Ni-NTA column (Qiagen, Hilden, Germany) as described previously in Mitchell and Gennis (33). Cyt*c*O and fluorescein-5-maleimide (F150; Invitrogen/Life Technologies/Thermo Fisher Scientific, Rochester, NY) were mixed in 100 mM HEPES pH 6.9, 0.1% DDM (Glycon Biochemicals, Luckenwalde, Germany) at 10 and 2 *μ*M, respectively, and incubated in the dark at room temperature (RT) for 15 min before free fluorescein was removed using a PD-10 column (GE Healthcare, Washington, NY) preequilibrated with 50 mM Tris pH 7.5 and 0.05% DDM. After concentration of the sample, protein concentration was determined and aliquots were frozen in liquid N_2_ and stored at −80°C until use.

### Growing and purification of membrane scaffold proteins in *Escherichia coli*

*E. coli* BL21 cells containing pMSP1E3D1 (12 nm disks) or pMSP1D1 (9 nm disks) (Addgene plasmids 20066 (34) and 20061 (35); https://www.addgene.org/) were grown at 37°C and 300 rpm in the presence of 30 *μ*g/mL of Kanamycin in LB and TB for precultures and expression cultures, respectively. Expression was induced with 1 mM IPTG at *OD*_600_ 2.5–3 and cells were harvested 3 h after induction.

His-tagged MSP1E3D1 and MSP1D1 were purified as described in Alvarez et al. (36) using Ni-NTA resin (Qiagen) and eluate was dialyzed against 20 mM Tris/HCl pH 8 and 100 mM NaCl. Purity was checked with sodium dodecyl sulfate-polyacrylamide gel electrophoresis and concentration was determined optically using *ε*_280_ 29,910 M^−1^ cm^−1^ and 21,430 M^−1^ cm^−1^ for MSP1E3D1 and MSP1D1 (37), respectively, before freezing in liquid N_2_ and stored at −80°C until use.

### Preparation of lipid nanodiscs

DOPG in chloroform was mixed with DOPE-Flu (1,2-dioleoyl-*sn*-glycero-3-phosphoethanolamine-*n*-(carboxyfluorescein)) at 1:30,000 fluorescent/nonfluorescent lipids in a round bottom flask and solvent was evaporated under a flow of N_2_. 50 mM Tris pH 7.5 and cholate was added to yield a final concentration at incubation of 20 and 26 mM for MSP1D1 and MSP1E3D1 NDs, respectively, and vortexed until lipids were dissolved. Using a low ratio of fluorescent over nonfluorescent lipids, the probability of having NDs with more than one labeled fluorophore can be neglected. Otherwise, with several fluorophores per ND, their blinking would partly cancel each other out and reduce the fluctuation amplitudes in the recorded FCS curves.

The lipid-cholate mixture were mixed with MSP protein at 10 mM lipids and 152 *μ*M MSP1D1 or 13 mM lipids and 100 *μ*M MSP1E3D1 to be in the optimal lipid and cholate concentration range to obtain NDs (37). After 1 h incubation at RT, 0.5–1 mL fractions were loaded onto preequilibrated PD-10 columns (50 mM Tris pH 7.5) and the eluted fractions containing NDs were pooled and concentrated to ∼120 *μ*L before being centrifuged at 10,000*g* for 20 min at 4°C. The sample was injected onto a Superdex 200 10/300 GL column (GE Healthcare) preequilibrated with 50 mM phosphate buffer pH 7.5 supplemented with 100 mM KCl at a flow rate of 0.25 mL/min. Elution was monitored optically at 280 nm, and fractions of 0.5 mL were collected. Typically, the elution chromatogram had two large peaks, with the void containing aggregates and the peaks containing the NDs; these peaks overlapped more or less. To ensure a sample without aggregates, fractions containing NDs were pooled, concentrated, and reinjected onto the Superdex 200 10/300 GL column (GE Healthcare) once or twice for the 9- and 12 nm NDs, respectively.

### Preparation of nanodiscs containing Flu-labeled Cyt*c*O

MSP/Cyt*c*O was kept constant at 120 and 12 nmol, respectively, and then mixed with a lipid-cholate mixture containing DOPG lipids dissolved in 50 mM Tris pH 7.5 to a final concentration at mixing of 5.3 mM lipids and 14 mM cholate for 9 nm NDs, and 10 mM lipids and 25 mM Cholate for 12 nm NDs. Samples were incubated for 1 h at RT and then treated as NDs containing only lipids. The chromatograms of the samples containing Cyt*c*O had a larger fraction of formed disks compared to the void/aggregated peak and samples were reinjected once onto the Superdex 200 10/300 GL column (GE Healthcare). Taken together, the probability that an ND contained a Cyt*c*O protein was found to be ≪1 (see remark in previous section), and as a result the eluted NDs fractions contained a mixture of NDs both with and without incorporated protein. However, only the NDs containing labeled Cyt*c*O can be observed and can contribute to the signal in the FCS measurements, and therefore these two populations were not further separated.

### Buffer exchange and sample handling

For pH measurements, the original phosphate buffer (50 mM pH 7.5 and 100 mM KCl) containing ND samples prepared as above was concentrated to a final volume of ∼500 *μ*L by using an Amicon Ultra-15 Centrifugal Filter Unit (Merck Millipore, Darmstadt, Germany) centrifuged at 4000×*g* and 4°C. Each concentrated ND sample was added to a PD-10 column (GE Healthcare), which was preequilibrated with 150 mM NaCl, followed by addition of 3.5 mL of 150-mM NaCl before being eluted with 3 mL of salt solution. For the Cyt*c*O in detergent solution, the 150 mM NaCl solution was supplemented with 0.05% of DDM. For buffer effect measurements, the original samples were aliquoted to at least two fractions, one for the phosphate and one for the HEPES buffer measurements. The finally prepared samples were bubbled with CO_2_-free synthetic air (AGA, Lidingö, Sweden) to remove CO_2_ dissolved in solution before measurements. Throughout the measurements the samples were then kept in a sealed container with continuous flow of CO_2_-free air. The pH was continuously monitored using a pH electrode (Inlab SemiMicro; Mettler-Toledo International, Columbus, OH) connected to a pH meter (SevenEasy pH meter S20; Mettler-Toledo International). The pH or the concentration of buffers was adjusted by additions of concentrated (0.1–0.5 M) NaOH/HCl or concentrated buffer solution (0.1 M) followed by three to four times of vortexing, each time for at least half a minute.

### FCS measurements and fitting

FCS measurements were performed on a home-built confocal setup (20, 21, 22), comprising an inverted microscope (Olympus model IX-70, Shinjuku, Tokyo, Japan) and a linearly polarized Ar ion laser (LGK 7812-1; Siemens, München, Germany) with emission at 488 nm and focused by a 40× NA, UPlanApo objective (Olympus). The laser beam radius in the focal plane was ∼0.33 *μ*m. The fluorescence emission was collected by the same objective, projected onto a pinhole of 50 *μ*m in diameter by a 150 mm achromatic lens, split by a 50:50 beam splitter cube, passed through a pair of band-pass filters (HQ532/70; Chroma Technology, Rockingham, VT) and finally detected by two avalanche photodiodes (APDs, SPCMAQR-14/16; Perkin-Elmer Optoelectronics, Wellesley, MA). The APD signals were processed by an ALV-5000/E correlator (ALV, Langen, Germany) that generated a semi-log-scale correlation curve, integrating the recorded fluorescence intensity fluctuations over the measurement time, as described in Eq. 1. The excitation power was kept constant at 40 *μ*W throughout the experiments. The recorded correlation curves were analyzed using a Levenberg-Marquardt nonlinear least-square curve fitting algorithm (Origin 8; OriginLab, Northampton, MA). A model correlation function was used for the FCS curve fitting, assuming that the fluorescence intensity fluctuations are generated by (1) diffusion (of free fluorophores or of fluorophore-labeled NDs) into and out of the confocal detection volume, and (2) transitions of the fluorophores back and forth into three different dark states (19):

(1) G(τ)=1Tm∫0TmF(t)F(t+τ)dt1Tm∫0TmF2(t)dt={F(t)=〈F〉+δF(t)}=〈δF(t)δF(t+τ)〉〈F(t)〉2+1=1N(1−P−T−R)(1+ττD)−1(1+τβ2τD)−1/2×(1−P−T−R+Pe−t/τprot+Te−t/τT+Re−t/τR)+1.

Here, F(t) is the detected fluorescence intensity at a time *t*; *τ* is the correlation time; $T_{m}$ denotes the measurement time over which the fluorescence fluctuations are integrated; square brackets signify the time average; *τ*_*D*_ is the average translational diffusion time of the fluorescent species through the confocal detection volume; *N* is the mean number of fluorophores in the detection volume; and *β* is the relationship between the axial and lateral extension of the detection volume. The value *P* signifies the fraction of protonated fluorophores, and *τ*_prot_ = 1/*k*_prot_ is the proton relaxation time, where *k*_prot_ is the proton relaxation rate. For a one-step reversible protonation reaction in a nonbuffered water solution, *k*_prot_ is given by the sum of the deprotonation rate constant, *k*_off_, and the protonation rate, *k*_on_ = *κ*_on_[H^+^]_bulk_, of the fluorophore:

(2) $$k_{\text{prot}}=k_{\text{off}}+\kappa_{\text{on}}{\left[{\text{H}}^{+}\right]}_{\text{bulk}}.$$

Among the other dark states, one could be attributed to triplet state formation (with its population *T* and relaxation time *τ*_*T*_ in Eq. 1) (38), and the other most probably to redox state formation (39) of the fluorescein dyes (relative population *R* and relaxation time *τ*_*R*_ in Eq. 1). The kinetics of these states, as well as the diffusion properties of the free fluorophores/NDs, can be expected not to vary with pH and buffer concentrations. The FCS curves from each individual experiment (at different pH values or different buffer concentrations) could thus be fitted globally with the same diffusion time (*τ*_*D*_), triplet parameters (*T* and *τ*_*T*_), and parameters for the third dark state (*R* and *τ*_*R*_). Before each experiment, a sample of fluorescein at pH 7.5, at which pH it is protonated to a negligible extent, was measured for calibration purposes. The triplet parameters were found to be very stable during the course of the whole project, with *τ*_*T*_ varying between 1.4 and 1.5 *μ*s and *T* between 24 and 28%. Therefore, the boundary values for these parameters were also included in the global fit. In the FCS experiments the concentrations were adjusted so that the average number of free fluorophores, or labeled NDs, in the confocal detection volume, *N*, was between 1 and 10.

### Monte Carlo simulations

Monte Carlo simulations of the proton exchange dynamics were performed, based on a model (see Fig. 4 *A*) taking three major proton exchange pathways into account: proton exchange between the membrane and the bulk solution (I); proton migration along the membrane surface (II); and direct protonation of the membrane-bound fluorescein molecule by the bulk (III). In the simulations, the buffer concentration dependence of the sum of the protonation and deprotonation rates of the pathways II and III, corresponding to the parameter *k*_prot_ measured by FCS, was investigated. Details of the model and the Monte Carlo simulations are given in the Supporting Material.

## Results

### Influence of the size of the surrounding lipid membrane area on the protonation dynamics

FCS was used to investigate the protonation kinetics of fluorescein, attached either to a single lipid molecule or to Cyt*c*O, incorporated in 9- and 12-nm diameter NDs composed of DOPG lipids. For NDs without insertion of Cyt*c*O, fluorescein was directly attached to a lipid in the membrane; for the samples with Cyt*c*O inserted, the Cyt*c*O itself was labeled (see Materials and Methods). The effects on the protonation kinetics of the size of the NDs, changes in pH, as well as of incorporation of Cyt*c*O in the NDs, were studied. The observed protonation kinetics was compared to that of free fluorophores and fluorophores attached to solubilized Cyt*c*O. In the following, to simplify notation, free fluorescein is denoted “flu” and “Cyt*c*O-flu” when coupled to solubilized Cyt*c*O. NDs with sizes of 9 and 12 nm are denoted, respectively, as “ND(9)” and “ND(12)”, and those with fluorescein as “ND(9/12)-flu”. ND samples with fluorescein-coupled Cyt*c*O inserted are referred to as “ND(9/12)-Cyt*c*O-flu”. For each sample category, up to three independent experiments were carried out, at ∼10 different pH values (see Materials and Methods for further details).

**Figure 1:**
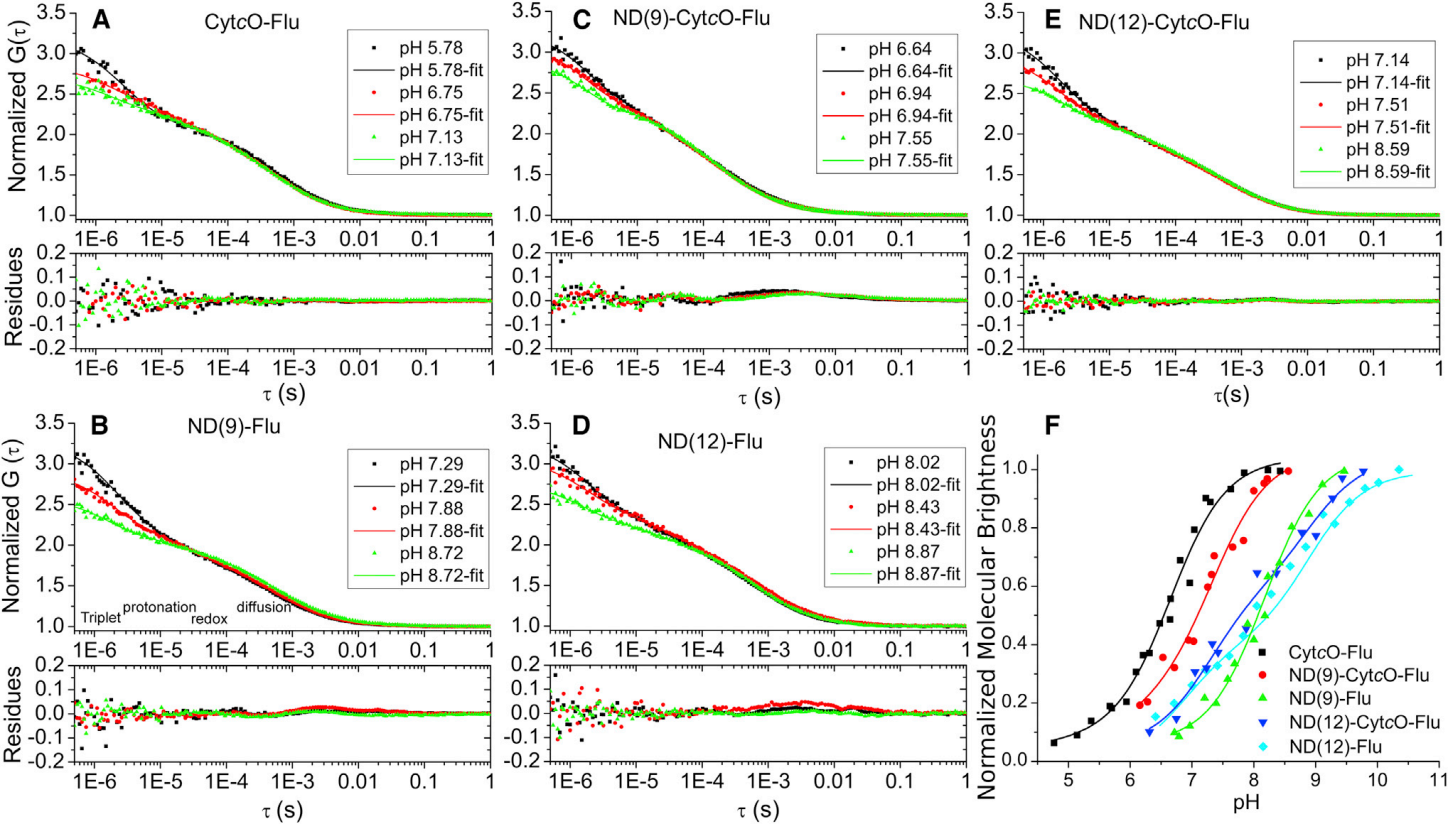
Normalized FCS curves (with *N* set to unity), recorded at different pH and from the different ND samples. The curves were recorded at pH values in parity with, or higher than, the p*K*_*a*_ values of fluorescein in the different samples, in a pH range where the membrane has been previously found to be fully active as a proton-collecting antenna (21). The curves were fitted to Eq. 1 (*solid lines*), with the fitting residuals given below the curves. (*A*) Fluorescein-labeled Cyt*c*O in detergent solution, Cyt*c*O-flu. (*B*) Fluorescein attached directly to DOPG NDs with a diameter of 9 nm, ND(9)-flu. (*C*) Fluorescein-labeled Cyt*c*O incorporated into DOPG NDs with a diameter of 9 nm, ND(9)-Cyt*c*O-flu. (*D*) Fluorescein attached directly to DOPG NDs with a diameter of 12 nm, ND(12)-flu. (*E*) Fluorescein-labeled Cyt*c*O incorporated into DOPG NDs with a diameter of 12 nm, ND(12)-Cyt*c*O-flu. (*F*) Molecular brightness of fluorescein at different pH values, as determined from FCS experiments, and with the pH titration curves fitted to Eq. 3. Cyt*c*O-flu (*black*), p*K*_a_ (1) = 5.0 (3.5% of the total amplitude) and p*K*_a_ (2) = 6.6 (96.5% of the total amplitude); ND(9)-Cyt*c*O-flu (*red*), p*K*_a_ (1) = 6.0 (14.5% of the total amplitude) and p*K*_a_ (2) = 7.2 (85.5% of the total amplitude); ND(9)-flu (*green*), p*K*_a_ (1) = 7.3 (10% of the total amplitude) and p*K*_a_ (2) = 8.1 (90% of the total amplitude); ND(12)-Cyt*c*O-flu (*blue*), p*K*_a_ (1) = 7.3 (52% of the total amplitude) and p*K*_a_ (2) = 8.8 (48% of the total amplitude); and ND(12)-flu (*cyan*), p*K*_a_ (1) = 6.8 (41% of the total amplitude) and p*K*_a_ (2) = 8.7 (59% of the total amplitude). To see this figure in color, go online.

First, the protonation kinetics of lipid-labeled fluorophores in NDs was investigated and the results were compared to those of free flu and Cyt*c*O-flu in solution. The reference measurements of the protonation kinetics of Cyt*c*O-flu (Fig. 1 *A*) displayed no significant differences to those of free flu, and were in agreement with previous studies by FCS (18, 20, 22). In contrast, for ND(12)-flu a shift in the pH dependence of the recorded FCS curves could be noticed (Fig. 1 *D*). Similar protonation (P) and protonation relaxation times (*τ*_prot_) were found for ND(12)-flu as for flu and Cyt*c*O-flu in solution, but at ∼100 times higher proton concentrations. This indicates a marked increase in the protonation rate for ND(12)-flu relative to that for flu and Cyt*c*O-flu. The increase is comparable to that previously observed in 30-nm-diameter unilamellar vesicles (SUVs) (20). Also, for ND(9)-flu a similar increase in the protonation rates could be observed (Fig. 1 *B*). ND(12)-flu and ND(9)-flu, with the dyes directly incorporated into the lipid membranes of the NDs, thus provide sufficiently large planar membrane-water interface areas (∼110 and 64 nm^2^, respectively) to effectuate the same enhancement of the protonation rate as that observed for 30-nm diameter SUVs (with an approximate area of 2800 nm^2^, outer surface). The rate constants are summarized in Table 1.

Next, we studied the protonation kinetics with the label attached to Cyt*c*O, and with the Cyt*c*O reconstituted into the NDs. For ND(12)-Cyt*c*O-flu the protonation kinetics, as observed in the FCS curves (Fig. 1 *E*), were found to be similar to those of ND(9)-flu and ND(12)-flu (Fig. 1, *B* and *D*, and Table 1). In contrast, with Cyt*c*O reconstituted into the smaller NDs, i.e., for ND(9)-Cyt*c*O-flu, the protonation rates were significantly lower (Fig. 1 *C*), and similar to those recorded for free flu and Cyt*c*O-flu in solution (Fig. 1 *A*).

As additional, independent information reflecting the protonation state of the fluorophores, the pH dependence of the fluorescein molecular brightness was also determined for all the samples mentioned above (Fig. 1 *F*). The molecular brightness of fluorescein was extracted by fitting the FCS curves to Eq. 1, and by dividing the recorded average fluorescence intensity with the average number of actively fluorescing fluorophore-labeled NDs present in the FCS observation volume (〈*F*(*t*)〉/*N*(1-*T*-*P*-*R*)). Fluorescein contains two protonatable groups (22). An equation (40) corresponding to the titration of a compound with two p*K*_*a*_ values was therefore fitted to the pH titration curves of the normalized molecular brightness (*NMB*):

(3) $$NMB=\frac{a}{1+10^{pK_{a}\left(1\right)-pH}}+\frac{1-a}{1+10^{pK_{a}\left(2\right)-pH}}+c.$$

Here, *a* denotes the amplitude for p*K*_*a*_ (1), and *c* is the offset.

The titrated NMB values for the different samples as a function of pH, together with the fitted titration curves for each sample (Fig. 1 *F*) are in agreement with the trend seen in the protonation-dependent part of the FCS curves (Fig. 1, *A*–*E*): the p*K*_*a*_ values of ND(9)-Cyt*c*O-flu were closer to the case of Cyt*c*O-flu in absence of lipid membranes, while the p*K*_*a*_ values of ND(12)-Cyt*c*O-flu were more comparable to those of ND(9)-flu and ND(12)-flu.

**Figure 2:**
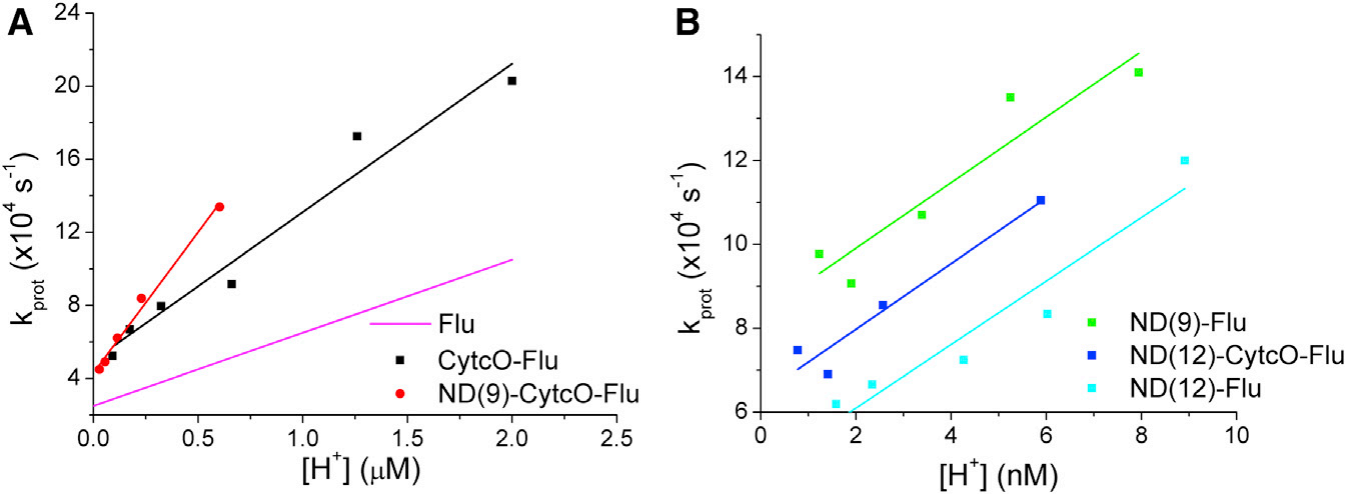
Protonation relaxation rates of fluorescein, *k*_prot_, as retrieved from FCS experiments, and as a function of bulk proton concentration, [H^+^]. (*A*) *k*_prot_ versus [H^+^] for Cyt*c*O-fluorescein (*black*), ND(9)-Cyt*c*O-fluorescein (*red*), and free fluorescein (*magenta*, curve made by taking values from Widengren et al. (18)). (*B*) *k*_prot_ versus [H^+^] for ND (9)-fluorescein (*green*), ND(12)-Cyt*c*O-fluorescein (*blue*), and ND(12)-Cyt*c*O-fluorescein (*cyan*). The proton on- and off-rates extracted from two to three independent experiments for each case done in this article and from reference are given in Table 1. To see this figure in color, go online.

From the FCS measurements, protonation on- (*κ*_on_, slope) and off- (*k*_off_, intercept) rates, can also be determined by performing a linear fit of the measured total protonation rate, *k*_prot_ (inverse of protonation relaxation time, *τ*_prot_) versus [H^+^], as shown in Fig. 2. In experiments with fluorophore-labeled SUVs, it was previously found that the protonation kinetics of the fluorophores display two regimes (21): at high pH (>8), where the membrane is fully active as a proton collecting antenna, and at low pH (<7), where direct protonation from the bulk solution dominates. To separately analyze the [H^+^] dependencies of *k*_prot_ in the high- and low-pH regimes, the fits for the ND samples were performed for pH values in parity with or higher than the p*K*_*a*_ values of fluorescein in these samples, as given by the plots in Fig. 1 *E*. Fig. 2 *B* shows a plot of *k*_prot_ versus [H^+^] obtained from ND(9)-flu, ND(12)-Cyt*c*O-flu and ND (12)-flu in the [H^+^] range 1–10 nM (pH 9–8). From the data and the corresponding linear fits, the slopes were found to be comparable between these samples. For ND(9)-Cyt*c*O-flu, the fraction of protonated fluorophores (*P* in Eq. 1) is too low to allow detection of *k*_prot_ in the same [H^+^] range. Within a slightly higher concentration range (>20 nM) however, the fitted slope of the *k*_prot_ versus [H^+^] data of ND(9)-Cyt*c*O-flu was found to be in a similar range as that for the Cyt*c*O-flu data (Fig. 2 *A*), with the slope for ND(9)-Cyt*c*O-flu approximately a factor of two steeper than that of Cyt*c*O-flu. Average values and standard deviations for the protonation on- and off-rates for all samples, determined from linear plots as shown in Fig. 2 are summarized in Table 1. The data show that the protonation on-rates for ND(9)-flu, ND(12)-CytcO-flu, and ND(12)-flu are almost two orders of magnitude higher than those for ND(9)-CytcO-flu and CytcO-flu and flu. In contrast, no significant differences in the protonation off-rates could be observed.

### Effects of ambient buffers in the bulk on the protonation kinetics at the membrane-water interface

**Figure 3:**
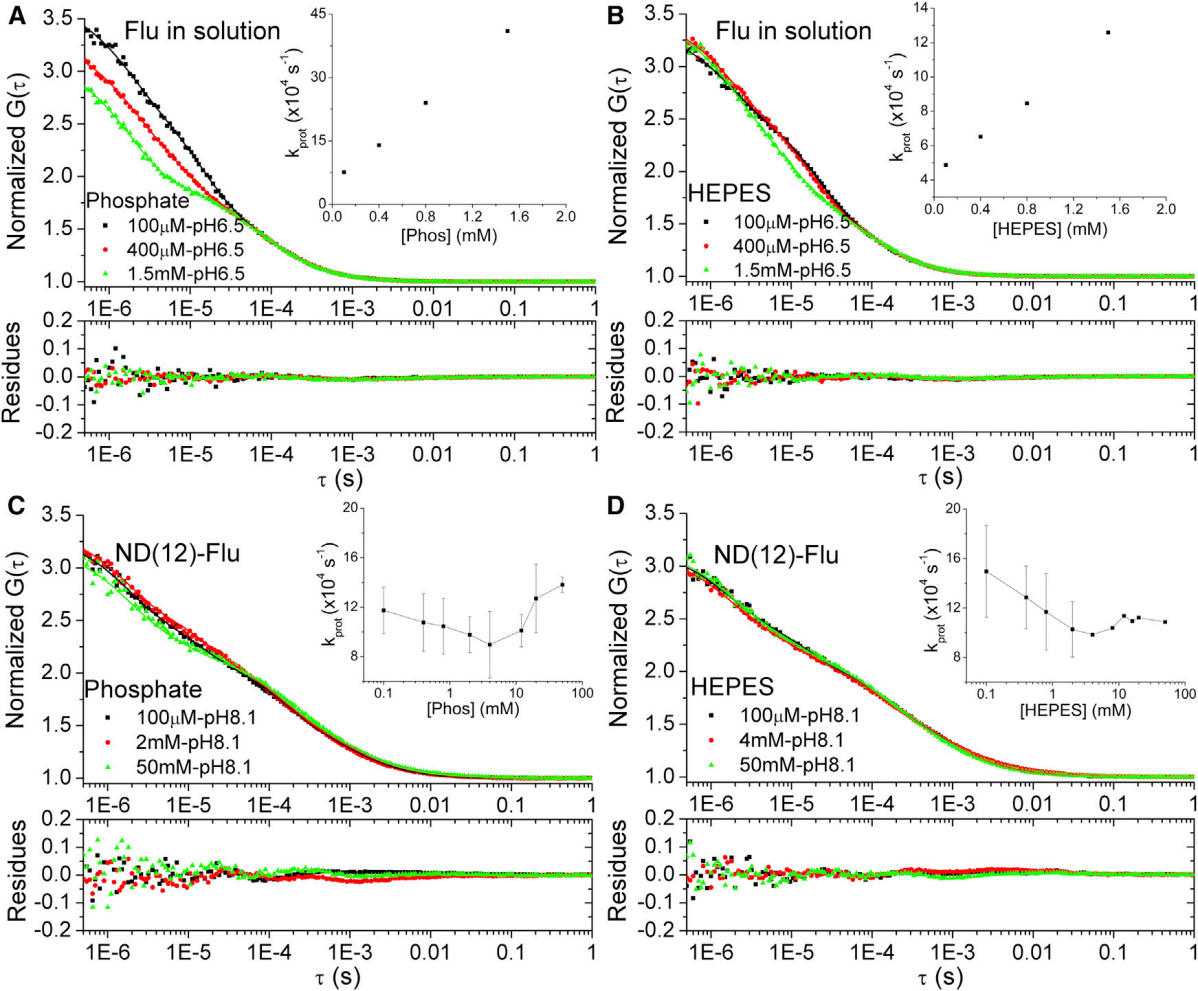
Normalized fluorescence correlation curves recorded from free fluorescein and from ND(12)-flu at different buffer concentrations. The curves were fitted to Eq. 1 (*solid lines*), with the fitting residuals given below the curves. (*A*) Fluorescein in phosphate buffer at pH 6.5. (*B*) Fluorescein in HEPES buffer at pH 6.5. (*C*) ND(12)-flu in phosphate buffer at pH 8.1. (*D*) ND (12)-flu in HEPES buffer at pH 8. To see this figure in color, go online.

The effects of external buffers (phosphate and HEPES, pH 8.1, concentrations 100 *μ*M to 50 mM) on the protonation kinetics of fluorescein at the surface of a lipid membrane was investigated using ND(12)-flu. As a reference, measurements were also done with free fluorescein in the same buffers (pH 6.5). The pH for the different samples was set close to the p*k*_*a*_ value of the fluorescein molecule in that sample, and still within the buffering range of the chosen buffers. The pH was therefore set differently for the control and the test samples due to the lower p*K*_*a*_ value of fluorescein in solution, compared to when attached to lipid membranes. Representative FCS curves measured at three chosen buffer concentrations for each sample as well as the dependence of the obtained total proton exchange rates, *k*_prot_, on the buffer concentrations are shown in Fig. 3. For free fluorescein, a pronounced enhancement of *k*_prot_ could be observed already at sub-mM buffer concentrations, with a stronger enhancement effect from the phosphate buffer (*inset*, Fig. 3 *A*) than from the HEPES buffer (*inset*, Fig. 3 *B*). The increase of *k*_*prot*_ displayed a linear dependence to the buffer concentrations, in agreement with results from previous studies (18). In contrast, for the ND(12)-flu sample, a different and more complex buffer dependence for *k*_prot_ was found (*insets*, Fig. 3, *C* and *D*). With phosphate buffer added in increasing concentrations to the ND(12)-flu sample, *k*_prot_ was first (for concentrations from 0.1 to ∼4 mM) found to decrease and thereafter to increase (for buffer concentrations above 5 mM). For ND(12)-flu in HEPES buffer, a similar dependence of *k*_prot_ was found, but with a less pronounced increase in *k*_prot_ with higher (>5 mM) buffer concentrations.

## Discussion

The results show that the area of the membrane-water interface surrounding a protein determines the efficiency of the proton-collecting antenna effect. For ND(9)-Cyt*c*O-flu, both the pKa values and the protonation on-rates were comparable to the values for Cyt*c*O-flu in aqueous solution, i.e., for the case when the protein is not surrounded by a lipid membrane (albeit detergent molecules in a micellar arrangement). In contrast, for ND(12)-Cyt*c*O-flu, providing a larger embedding membrane area for Cyt*c*O-flu, a significant enhancement of the protonation rates was observed, reflected both in the pKa values and in the protonation exchange rates. Interestingly, for the protonation kinetics of ND(12)-flu, ND(12)-Cyt*c*O-flu), and ND(9)-flu, no significant differences could be observed, and the kinetics was also very similar to what has been previously reported for 30–40 nm diameter SUVs composed of DOPG lipids (*κ*_on_ = 9.4 × 10^12^ s^−1^ M^−1^) (20). Given that the diameter of the *R. sphaeroides* (aa3) Cyt*c*O is ∼6.5 nm (41), the lipid membrane area in ND(12)-Cyt*c*O-flu and in ND(9)-Cyt*c*O-flu is ∼80 and 30 nm^2^, respectively. Thus, the data indicate that the proton-collecting antenna effect saturates already for a membrane area of *π*(9 nm/2)^2^ ∼60 nm^2^ (ND(9)-flu), i.e., a lipid area in the range of 60–80 nm^2^ is sufficient to effectuate a full enhancement of the protonation rates. Correspondingly, a major reason for the small membrane proton-collecting antenna effect observed for ND(9)-Cyt*c*O-flu (only a difference in the on-rate by a factor of two compared to that of Cyt*c*O-flu) is presumably the limited remaining space for lipid molecules in the 9 nm NDs when Cyt*c*O-flu is inserted, i.e., a lipid area of ∼30 nm^2^ is apparently not sufficient to support a strong enhancement of the protonation rate. It can be noted that in our previous publication (22), where Cyt*c*O-flu instead of fluorescein-labeled lipids was inserted into the same type of SUVs, a protonation on-rate *κ*_on_ = (3.1 ± 0.4) × 10^13^ s^−1^ M^−1^ was reported, a factor of four larger than that determined for ND(12)-Cyt*c*O-flu in this study. This finding may indicate that interactions between the protein and a membrane of sufficiently large area may further enhance the protonation rate. Nevertheless, when the membrane area increases from ∼30 nm^2^ (ND(9)-Cyt*c*O-flu) to ∼80 nm^2^ (ND(12)-Cyt*c*O-flu) the *κ*_on_ rate increases by a factor of 50, but then only with an additional factor of 4 when the membrane area is further increased to ∼2800 nm^2^ (Cyt*c*O in 30-nm diameter SUVs) (22). This indicates that the surrounding membrane area has a prominent influence on the protonation, with a limiting area for its enhancement in the range of 60–80 nm^2^, given the saturation of the proton collecting antenna in the case for ND(9)-flu (∼60 nm^2^) without the incorporation of Cyt*c*O.

This range for the limiting area indicates that the corresponding *R*_PCA_ would be as small as 4–5 nm (ND(9)-flu). This is approximately three orders of magnitude smaller than the range of *R*_*PCA*_ derived from long-range proton exchange at membrane-water interfaces (15). This may however be understood from the broad range of *D*_*s*_ reported, indicating a time- and length-scale dependent proton diffusion (27, 28). *R*_PCA_ can approximately be related to the mean-square displacement of a proton along the membrane surface during its average dwell time, *τ*_*s*_, on the same surface. For 2D diffusion, this yields:

(4) $$R_{\text{PCA}}∼2\sqrt{\frac{D_{s}\tau_{s}}{\pi }}.$$

Apart from the two orders-of-magnitude higher *D*_*s*_ measured for long-range proton exchange (1, 12, 25), compared to the *D*_*s*_ for local, steady-state exchange, as studied by FCS (20), long-range proton transfer is likely to be a consequence of the longer effective dwell times, *τ*_*s*_, following from thousands of desorption and reabsorption cycles at the surface by the protons (1). In view of Eq. 4, the significantly larger *D*_*s*_ and longer *τ*_*s*_ expected for long-range proton transfer than for the local exchange can thus well explain the much smaller *R*_PCA_ values found in this study.

**Figure 4:**
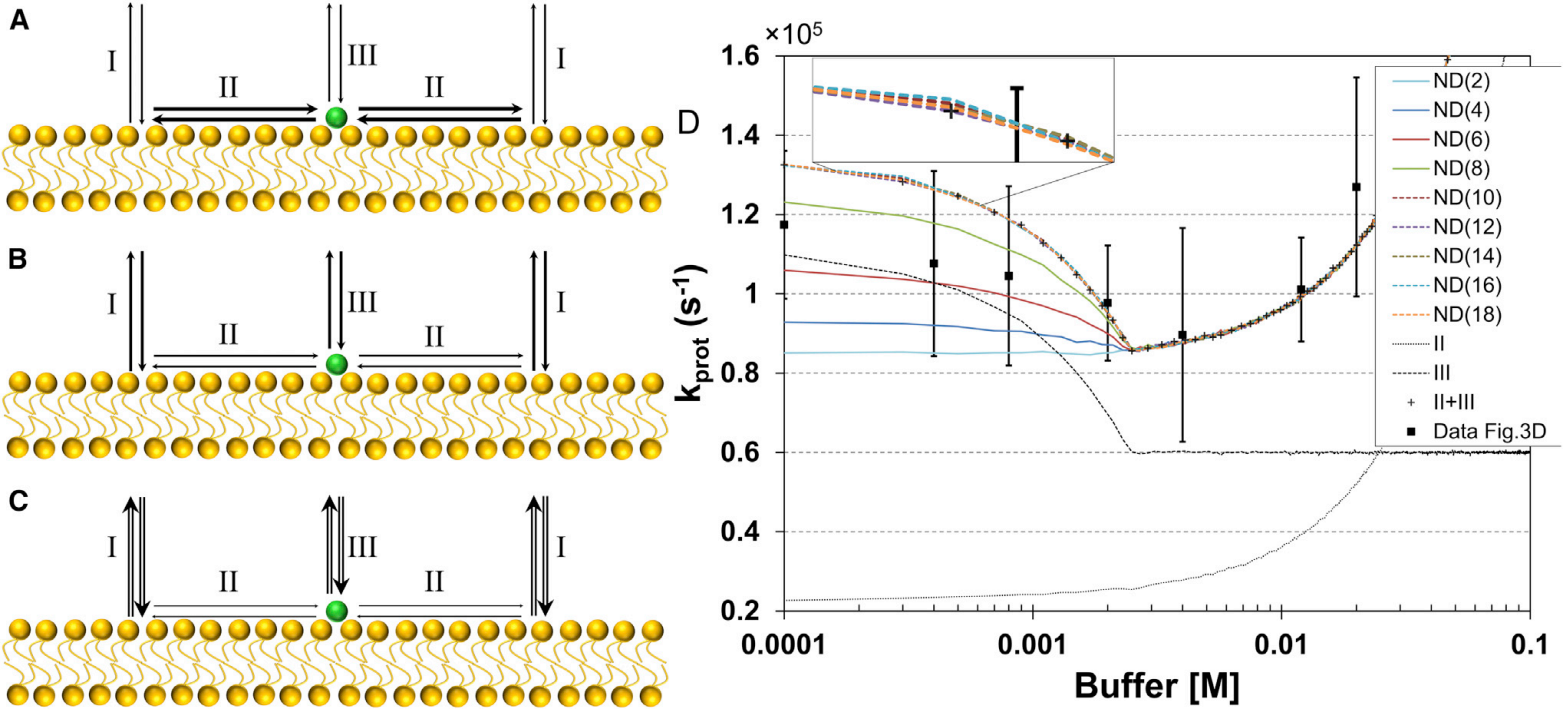
Proposed mechanism for the observed membrane protonation dynamics dependence on the bulk buffer concentration. Three major proton exchange pathways are considered, i.e., proton exchange between the membrane and the bulk solution (I), proton migration along membrane surface with subsequent proton exchange between the surface and the fluorophore (II), and direct proton exchange between the membrane bound fluorescein molecule and the bulk (III). Only the sum of the protonation relaxation rates of the pathways II and III are accessible by FCS measurements. Thickness of arrows represents the magnitude of the proton exchange rates. (*A*) The proton exchange rates at low buffer concentrations (<1 mM). (*B*) Proton exchange rates at medium buffer concentrations (∼4 mM). (*C*) Proton exchange rates at high buffer concentrations (>10 mM). (*D*) Monte Carlo simulations of the phosphate buffer concentration dependence of *k*_prot_ for fluorescein-labeled NDs of different diameters (see the Supporting Material for further details and parameter values used in the simulations). Apart from the overall dependence of the experimentally accessible protonation relaxation rate *k*_prot_ = (II+III), the dependence of the protonation relaxation rates of the individual pathways II and III on the bulk buffer concentration is also shown. The differences in the simulated curves for ND (10)-ND (18) are so small that they would not be experimentally discernible (*magnified inset*). (*Black squares*) Experimental data for *k*_prot_ for ND (12)-flu (from *inset* of Fig. 3*C*), with standard deviations given by the error bars. To see this figure in color, go online.

Given that the proton diffusion along membranes can be considered anomalous and time- and length-scale dependent, one can also expect the effects of added buffers to be different for the local and the long-range proton exchange. For long-range proton transfer, membrane-bound as well as bulk buffers significantly reduce *D*_*s*_ (13, 14, 25, 30), in proportion to the fraction of protonated buffer molecules and by their limited diffusivity compared to *D*_*w*_ (23, 29). This leads to significantly smaller *R*_PCA_ values, already at mM buffer concentrations (11, 15, 31). For the local proton exchange at steady state, as observed by FCS, a prominent linear increase in the protonation rate can be observed with increasing concentrations of membrane-bound buffer molecules (20). This is likely a consequence of an increased local concentration of protons at the membrane-water interface, and that the diffusion coefficients of the buffering molecules (lipids in Brändén et al. (20)) are almost in parity with the relatively low, local *D*_*s*_. For a mobile buffer, as studied here, the local protonation rate shows a yet different buffer concentration dependence. The found dependence (Fig. 3, insets of *C* and *D*), with *k*_prot_ first decreasing with increasing buffer concentrations, and then remaining constant or slightly increasing at concentrations >3–5 mM, can be explained from a simple kinetic model (20, 21) by considering that higher buffer concentrations in the ambient solution of the membrane surface not only increase the proton exchange between the membrane-bound dye and the solution, but also the exchange between the membrane surface as a whole and the solution. (see Fig. 4, *A*–*C*, for reference). At low buffer concentrations in the bulk (Fig. 4 *A*), in our case at buffer concentrations below 1mM, the proton exchange between the dye and the membrane surface (II) is higher than that between the dye and the bulk solution (III). Increased buffer concentrations (Fig. 4 *B*) primarily promote the proton exchange between the membrane surface and the bulk (I). The typical distance that protons can migrate along the membrane surface before they are dissipated form the surface will therefore decrease. Like for long-range proton transfer, increased buffer concentrations will thus make the effective area of the proton-collecting antennae of the membrane-bound fluorophore smaller, and reduce the exchange between the membrane surface and the dye (II). There will also be an increase in the direct proton exchange between the buffer and the membrane-bound dye (III), but because this increase is smaller than the decrease in (II) caused by the promoted proton exchange between the membrane surface and the bulk, there will be an overall decrease in *k*_prot_ for the dye. At further increased buffer concentrations (Fig. 4 *C*), in our case at concentrations >5 mM, the direct proton exchange between the dye and the bulk (III) increases correspondingly, while the protonation of the dye via the membrane surface (II) is further diminished and becomes negligible. As a consequence, *k*_prot_ then starts to increase with higher buffer concentrations.

To test this explanation of how *k*_prot_ depends on the mobile buffer concentration (Fig. 3, *C* and *D*), and if it can also incorporate the surrounding membrane area dependence, Monte Carlo simulations of the proton exchange were performed. In the simulations, the proton exchange pathways I–III were considered for fluorophore-labeled NDs with different diameters, assuming the fluorophore to be located in the center of the ND, and proton migration along the surface to occur as for 2D diffusion, with a diffusion coefficient of *D*_*s*_ = 2 × 10^−7^ cm^2^/s (20). With this value for *D*_*s*_, with a *R*_PCA_ of ∼5 nm, and use of Eq. 4 gives us an estimate of *τ*_*s*_ of ∼1 *μ*s. The inverse value of *τ*_*s*_ was used in the simulations as an estimate of the proton dissociation rate from the membranes. The other rate parameter values used in the simulations were based on experimentally determined values in this study, or in previous works (18, 20). Further details are given in the Supplementary Information. From the outcome of the simulations (the phosphate buffer concentration dependence of *k*_prot_ for ND(12)-flu is shown in Fig. 4 *D*, and the corresponding dependence for the HEPES buffer concentration in Fig. S1 in the Supporting Material), it can be seen that they indeed can reproduce the experimentally observed buffer concentration dependence (Fig. 3, *C* and *D*). By considering the dependence of the proton exchange pathways II and III separately, it can be noticed that protonation of the dye via the membrane surface (II) gets fully suppressed, while direct exchange with the bulk increases linearly, with increasing bulk buffer concentrations. The simulations and the model used can also reproduce the observed effects of the ND surface area, with no essential enhancement of *k*_prot_ for ND diameters larger than 10 nm. Independent of the ND diameter however, the minimum overall protonation rate, *k*_prot_, are obtained at the same buffer concentration (in our simulations, as well as in the experiments, ∼2–4 mM).

In the study of the buffer concentration dependence, a difference between buffers was also obvious. For the case of free fluorescein, the phosphate buffer promotes *k*_prot_ more strongly than the HEPES buffer (Fig. 3, *A* and *B*), which is also a known effect (18). One possible explanation is the larger size of the HEPES molecules compared to that of the phosphate, which decreases the collisional rates underlying the proton exchange. The larger size of the HEPES molecules, and a thereby lowered ability to reach into the membrane-water interface, is also most likely a major reason for the smaller effects of the HEPES buffer on the proton exchange rates for the ND(12)-flu sample, as could be seen experimentally (Fig. 3, *C* and *D*) and which was further supported by the simulations (Figs. 4 *D* and S1).

In summary, our investigations of the local membrane proton-collecting antenna effect show that ND(9)-flu, ND(12)-flu, and ND(12)-CytcO-flu, with estimated associated membrane areas of 64, 80 and 110 nm^2^, display similarly enhanced *κ*_on_ rates, enhanced to the same level as the *κ*_on_ rates found for 30-nm diameter vesicles (∼2800 nm^2^ outer surface area) (20). Beyond a planar membrane-water interface of ∼60 nm^2^ no major additional increase in κ_on_ is observed, and this area thus seems to be sufficient to effectuate a full enhancement of the protonation of membrane-bound protonatable fluorophores. In contrast, the interface area of ND(9)-CytcO-flu (∼30 nm^2^) is clearly insufficient, and yields a *κ*_on_ rate very close to those found for CytcO-flu and Flu. The bulk buffer concentration dependence of *k*_prot_ was found to strongly deviate from a linear dependence, previously observed when both fluorophores and buffers are free in solution or when both are confined to the membrane (18, 20).

It is difficult to estimate the buffer concentration near the membrane at physiological conditions. The relevant parameter is the concentration of free buffer near, e.g., the mitochondrial membrane, which most likely significantly deviates from the overall buffering capacity. This is because the volume is relatively densely packed with, e.g., proteins and the collective buffering capacity is determined also by the protein surface groups, which are present at high concentrations. The ionic strength in the mitochondrial intermembrane space has been found to be approximately the same as that of the cytosol, i.e., 100–150 mM (42), but only a small fraction of these ions contribute to the buffering capacity. Nevertheless, we note that the buffer concentration interval at which we found the reported effects (1–10 mM), corresponds to that of phosphate in the cytoplasm (∼10 mM).

The observed dependencies of *k*_prot_ on the ND area and the buffer concentration are different from that previously reported for long-range proton transfer (13, 14, 25, 30), but can be explained by the quite different proton diffusion coefficients and surface dwell times found on a local scale, compared to when considered over hundreds of micrometers over the membranes. The *D*_*s*_ recorded over longer spatial scales (12, 13, 14) can be expected to represent an average of the diffusion coefficients for the protons (11, 23, 29), weighted by the relative fractions of protons: (1) in bulk water close to the membrane, and not interacting with buffer molecules; (2) bound to buffer molecules; and (3) at the membrane-water interface. In contrast, what we observe in the FCS measurements (20) are likely the diffusion properties of the protons in condition (3). For this condition, MD simulations indicate two major diffusion modes of the protons (27, 28): (3-A), a slower mode, where hydronium ions would be tightly bound to the lipids (27), or where protons would be trapped within the lipid headgroup region (28), and the diffusion of the protons in this mode would then correspond to the lipid diffusion; and (3-B), a second mode, with protons in small water clusters, less tightly bound within the lipid headgroup region, occasionally jumping from one cluster to another (27), or with the protons confined in the smaller interface region between the membrane and the bulk water (28). In both scenarios, the protons in this mode would display a slightly faster diffusion than in mode (3-A). Although with different detailed mechanisms suggested, both MD simulation studies (27, 28) indicate that jumps between small water clusters, and switching between the modes (3-A) and (3-B), take place on a nanosecond timescale. Numerous switching cycles would then occur within an estimated *τ*_*s*_ ∼1 *μ*s, and the *D*_*s*_ determined by FCS would thus represent an average of the proton diffusion properties in modes (3-A) and (3-B). The determined *D*_*s*_ (20) is 2–3 times faster than for lipids (24), which is well in line with the expected average diffusion behavior of (3-A) and (3-B), and with the results in Wolf et al. (27) and Yamashita and Voth (28). The long-range (tens of micrometers) proton transfer along biological membranes (12, 13, 14) has been suggested to occur via thousands of desorption and reabsorption cycles (1). Our results are well in line with this view, and our estimated *τ*_*s*_ would then represent an average dwell time of a proton in state (3) within one such cycle. The above interpretation was further supported by Monte Carlo simulations, based on a simple kinetic model, and on parameter values determined by FCS. The experimentally observed dependencies of *k*_prot_ on the ND area and the buffer concentration could then be fully regenerated. Taken together, this study supports a unified view of experimental data from long-range (12, 13, 14) and local-scale proton exchange studies (20, 21, 22) at biological membranes. Further, it confirms recent theoretical work (27, 28) concluding that the efficiency and buffer dependence of the proton-collecting antenna effect is time- and length-scale dependent, reflecting the anomalous character of proton diffusion along membranes. This study provides a good starting point for further FCS studies of the detailed proton exchange and diffusion mechanisms at biological membranes, as predicted by theoretical work, as well as for studies of these mechanisms at mitochondrial membranes, on isolated mitochondria or in live cells.

## Author Contributions

P.B. and J.W. designed research; L.X. and L.N.Ö. performed experiments; J.B., P.B., L.X., and J.W. analyzed data; and L.X. and J.W. wrote the article.

## Figures and Tables

**Table 1:** Protonation and Deprotonation Rates of Fluorescein under Different Conditions

	Flu in Water (18, 20)	Cyt*c*O-Flu	ND(9)-Cyt*c*O-flu	ND(9)-Flu	ND(12)-Cyt*c*O-flu	ND(12)-Flu
*κ*_on_ (M^−1^ s^−1^)	4 × 10^10^	(1.01 ± 0.27) × 10^11^	(1.44 ± 0.24) × 10^11^	(7.54 ± 0.44) × 10^12^	(7.71 ± 0.54) × 10^12^	(7.35 ± 0.33) × 10^12^
*k*_off_ (s^−1^)	2.5 × 10^4^	(6.85 ± 2.63) × 10^4^	(4.66 ± 0.33) × 10^4^	(6.72 ± 2.28) × 10^4^	(6.99 ± 0.66) × 10^4^	(5.28 ± 0.99) × 10^4^

## Supporting Material

Document S1. Supporting Materials and Methods, descriptions of Monte-Carlo simulation algorithm, one figure, and one table

Document S2. Article plus Supporting Material
