# Supplementary material for: Protonation Dynamics on Lipid Nanodiscs: Influence of the Membrane Surface Area and External Buffers
Source: Biophys J. 2016 May 10;110(9):1993–2003. doi: 10.1016/j.bpj.2016.03.035 (PMC4939474; doi:10.1016/j.bpj.2016.03.035)
Supplement: Document S1. Supporting Materials and Methods, descriptions of Monte-Carlo simulation algorithm, one figure, and one table [file mmc1.pdf]

Biophysical Journal, Volume *110*

Supplemental Information

Protonation Dynamics on Lipid Nanodiscs: Influence of the Membrane Surface Area and External Buffers

Lei Xu, Linda Näsvik Öjemyr, Jan Bergstrand, Peter Brzezinski, and Jerker Widengren

Supplementary Information

Protonation dynamics on lipid nanodiscs – influence of the membrane surface area and external buffers

L Xu, L Näsvik Öjemyr, J Bergstrand, P Brzezinski, J Widengren

Monte-Carlo simulations

Simulations of the proton exchange of fluorophore-labelled NDs with different diameters were analyzed, with the fluorophore located in the center of the ND. With reference to figure 4, three major proton exchange pathways were taken into consideration:

<u>I: Proton exchange between the membrane and the bulk solution</u>

$$BH + L^- \underset{k_{-1}}{\overset{k_{+1}}{\rightleftarrows}} LH + B^- , \qquad \text{(S1)}$$

where B^- and BH represent the base and acid forms of the buffer molecules, and L^- and LH are the base and acid forms of the lipid molecules in the membrane. At a certain pH, the equilibria between the acid and base forms of the buffer and lipid molecules are given by:

$$B^- + H^+ \underset{k_{H-1}}{\overset{k_{H+1}}{\rightleftarrows}} BH \qquad \text{(S2)}$$

$$L^- + H^+ \underset{k_{H-2}}{\overset{k_{H+2}}{\rightleftarrows}} LH \qquad \text{(S3)}$$

<u>II: Proton migration along the membrane surface, with subsequent proton exchange between the surface and the fluorophore</u>

$$LH + Fl^{2-} \underset{k_{-2}}{\overset{k_{+2}}{\rightleftarrows}} L^- + HFl^- , \qquad \text{(S4)}$$

where Fl^{2-} is the dianionic, fluorescent form of the fluorescein fluorophore and HFl^- is the protonated, practically non-fluorescent form of the fluorophore. At a certain pH, the equilibrium between the acid and base forms of the fluorophore molecules is given by:

$$Fl^{2-} + H^{+} \underset{k_{H-3}}{\overset{k_{H+3}}{\rightleftarrows}} HFl^{-} \tag{S5}$$

III: Direct proton exchange between the membrane bound fluorescein molecule and the buffer molecules in the bulk

$$BH + Fl^{2-} \underset{k_{-3}}{\overset{k_{+3}}{\rightleftarrows}} B^{-} + HFl^{-} \tag{S6}$$

In the simulations, we assume the proton migration along the membrane surface to occur as for two-dimensional diffusion. The protonation rate of the fluorophore can then be related to the probability for a proton undergoing 2D diffusion (with diffusion coefficient D_s) at the ND surface to reach a certain mean average displacement, r, within a certain time, t, expressed as

$$p(r)drd\theta = \frac{r}{D_s \cdot t} e^{-\frac{r^2}{D_s \cdot t}} drd\theta \tag{S7}$$

Monte Carlo Algorithm

Following equations S1-S7, Monte Carlo simulations were performed based on a program in MatLab. For Eq. S1-S6, the probability for each reaction was determined by multiplying each rate, *k*, with a small time increment, *dt*, such that $0<k\cdot dt<1$. This product can be interpreted as a probability for the reaction with rate constant *k* to occur within the time interval *dt*.

During a timestep a uniformly distributed random number between 0 and 1, *rand*, is generated for each of the reactions in Eq. S1,S3,S5 and S6, and for the k_{-2} reaction of Eq. S4. This number is compared with the probabilities, given by $k\cdot dt$. In the code, this was done by if-statements, i.e. if *rand*< $k\cdot dt$: then the reaction occur, else: it does not. If a reaction occurs resulting in the fluorophore being protonated (Eq. S4-S6) a counter for the protonation is increased by 1, i.e. *protonation = protonation+1*. Similarly, each time the fluorophore releases a proton (Eq. S4-S6) a counter for the deprotonation is increased by 1, i.e. *de_protonation = de_protonation+1*. Iterating over 10^7-10^9 time-steps, and for each iteration keeping track of which reactions occur, will yield the total protonation relaxation rate given by $k\_prot = (protonation+de\_protonation)/T_{sim}$, where $T_{sim} = dt\cdot$ (number of iterations), is the total simulation time (1-10s).

In the simulations, the fluorophore protonation via pathway II (Eq. S4), also requires the diffusion of protons along the lipid membrane to be considered. The probability for a proton to diffuse a distance *r* along the membrane during the time step *dt* is given by the solution of the 2D diffusion equation, as stated in Eq. S7. First however, for proton diffusion during a timestep to be relevant (at least) one lipid must be protonated. The probability for this is assumed to be proportional to the number of lipids in the ND and was estimated by Eq. S1 and S3 as the sum of $k_{+1}\cdot\pi(d/2)^2\cdot\ \rho\cdot$ [BH]$\cdot dt$ and $k_{H+2}\cdot\pi(d/2)^2\cdot\ \rho\cdot$ [H]$\cdot dt$, with *d* denoting the diameter of the ND and with the density of lipids, ρ, estimated to 4 nm^{-2}. Following the protonation of a lipid it can either be deprotonated by giving back the proton to the bulk. The probability for this is given by the sum of $k_{-1}\cdot[B^-]\cdot dt$ and $k_{H-2}\cdot dt$ (Eq. S1 and S3). In this case, the proton does not protonate the fluorophore. Alternatively, the proton diffuses along the membrane, and then encounters and protonates the fluorophore *(protonation = protonation+1*). With an initial distance between the fluorophore and a protonated lipid in the ND

area of R, the probability for a proton to diffuse a distance R or longer in a time dt is given by the integral of Eq. S7,

$$p(r > R) = \frac{2}{D_s \cdot dt}\int_R^\infty re^{-\frac{r^2}{D_s \cdot dt}}dr = e^{-\frac{R^2}{D_s \cdot dt}} \quad \text{(S8)}$$

The maximum distance a proton can travel along the surface of an ND is limited not only by the rates k_{-1} and k_{H-2} but also by the ND diameter, d. The average distance between protons on the membrane is estimated by the square root of the inverse of the proton density on the membrane, that is $R_{av} \sim \sqrt{A/(\pi N)}$,where N is the number of protons on the membrane (determined by the previous steps in the algorithm of protonation and deprotonation of lipids) and A is the area of the ND. The probability (given that one or more lipids are protonated) for a proton to diffuse to the fluorophore can then be expressed as

$$p(r \geq R_{av}) = e^{-\frac{R_{av}^2}{D \cdot dt}}. \quad \text{(S9)}$$

For small NDs, the case can be that $d/2<R_{av}$. In the simulations, we then set $R_{av} = d/2$.

Some of the rates are concentration dependent so that they have to be multiplied, not only with dt, but also with the concentration in order to get the probability. These rates are:

k_{-1} and k_{-3} (Eq. S1 resp. S6) depends on [B], k_{+1} and k_{+3} (Eq. S1 resp. S6) depends on [BH] and k_{H+2} and k_{H+3} (Eq. S3 resp. S5) depends on $[H^+]$.

The concentrations were calculated by the following equations

$$[H^+] = 10^{-pH} \quad \text{(S10)}$$

$$[B^-] = \frac{[B_{tot}]}{1 + \frac{[H^+]}{10^{-pK_a(buffer)}}} \quad \text{(S11)}$$

$$[BH] = \frac{[H^+][B_{tot}]}{[H^+] + 10^{-pK_a(buffer)}} \quad \text{(S12)}$$

where $[B_{tot}]$ is the total buffer concentration and $pK_a(buffer)=7.2$ (pK_a forphosphate buffer).

Table S1: For the simulations in figure 4D the following values of the various parameters were used.

Parameter	Value	Comment
dt	$1{\cdot}10^{-7}$ s	
pH	8.1	pH used in the ND measurements (Fig. 3C and 3D)
$[B_{tot}]$	0.001-0.1 M	Buffer concentrations used in the ND measurements (Fig. 3C and 3D)
pK_a of buffer	7.2	Parameter values for k_{H+1} and k_{H-1} (Eq. S2) are only included indirectly, via the pK_A value for the buffer and Eq. S10 and S11.
T_{sim}	5 s	
D_s	$2{\cdot}10^{-7}$ cm^2/s	From (18)
d (diameter of nano disc)	2-18 nm	
k_{+1}	$5{\cdot}10^8$/[L][BH]s	
k_{-1}	$5{\cdot}10^8$/[B]s	
k_{H-2}	$1{\cdot}10^6$/s	Estimated from Eq. 4, with D_s=$2{\cdot}10^{-7}$ cm^2/s and R_{PCA}=5µm.
k_{H+2}	$2{\cdot}10^3$/[L]s	pK_a(DOPG)=log[k_{H-2}/k_{H+2}] ≈2.7
k_{-2}	$4{\cdot}10^4$/s	The k_{off} values in table 1 for the ND samples correspond approximately to the sum of k_{-2} and k_{H-3}
k_{H+3}	$4{\cdot}10^{10}$/[H]s	From (17)
k_{H-3}	$2.5{\cdot}10^4$/s	From (17)
k_{+3}	$9{\cdot}10^5$/[BH]s	Estimated from the rate plot in Fig. 3B (inset) assuming a pK_a of phosphate of 7.2
k_{-3}	$6{\cdot}10^5$/[B]s	Estimated from the rate plot in Fig. 3B (inset) assuming a pK_a of phosphate of 7.2

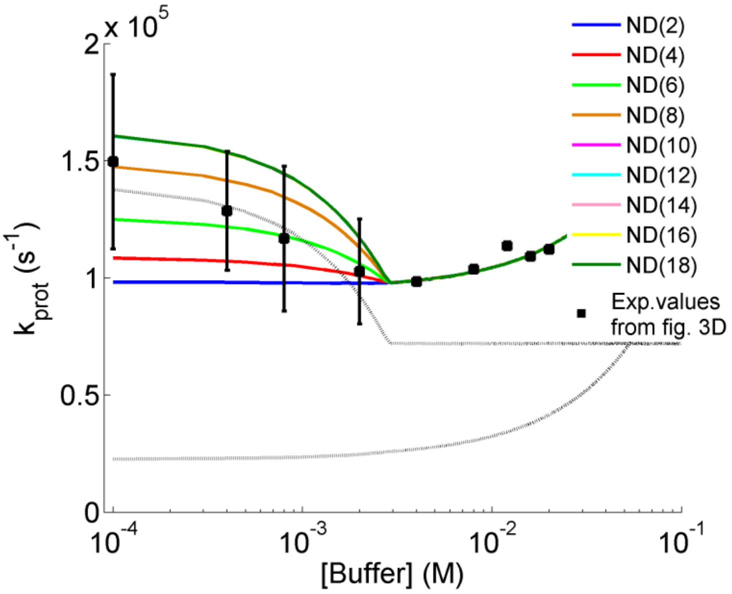

Figure S1: Monte-Carlo simulations of the HEPES buffer concentration dependence of k_{prot} for fluorescein-labelled NDs of different diameters. The same parameter values were used in the simulations as for the phosphate buffer simulations shown in figure 4D (see table S1 above), except for the buffer-related parameters k_{+3} (set to $k_{+3} = 1\cdot10^6$/[BH]s), k_{-3} (set to $k_{-3} = 9\cdot10^5$/[B]s) and the pK_a of buffer (set to 7.5). It can be noted that the set k_{+3} and k_{-3} values resulting in simulations which follow the experimental data are lower than the corresponding rate parameters used in the phosphate buffer simulations in figure 4D. This most likely reflects the larger size of the HEPES buffer molecules, their slower diffusion and lower accesibility to the membrane surface. Apart from the overall dependence of the experimentally accessible protonation relaxation rate k_{prot} = (II+III), the dependence of the protonation relaxation rates of the individual pathways II and III on the bulk buffer concentration is also shown. Black squares: experimental data for k_{prot} for ND(12)-flu (from inset of Fig. 3D), with standard error of the mean given by the error bars.
